# Cellular and Molecular Signatures of Oxidative Stress in Bronchial Epithelial Cell Models Injured by Cigarette Smoke Extract

**DOI:** 10.3390/ijms23031770

**Published:** 2022-02-04

**Authors:** Chiara Cipollina, Andreina Bruno, Salvatore Fasola, Marta Cristaldi, Bernardo Patella, Rosalinda Inguanta, Antonio Vilasi, Giuseppe Aiello, Stefania La Grutta, Claudia Torino, Elisabetta Pace

**Affiliations:** 1 Ri.MED Foundation, 90133 Palermo, Italy; ccipollina@fondazionerimed.com (C.C.); mcristaldi@fondazionerimed.com (M.C.); 2 Institute for Biomedical Research and Innovation, National Research Council, 90146 Palermo, Italy; andreina.bruno@irib.cnr.it (A.B.); salvatore.fasola@irib.cnr.it (S.F.); stefania.lagrutta@irib.cnr.it (S.L.G.); elisabetta.pace@irib.cnr.it (E.P.); 3 Institute of Translational Pharmacology, National Research Council, 90146 Palermo, Italy; 4 Department of Engineering, University of Palermo, 90128 Palermo, Italy; bernardo.patella@unipa.it (B.P.); rosalinda.inguanta@unipa.it (R.I.); giuseppe.aiello03@unipa.it (G.A.); 5 Institute of Clinical Physiology, National Research Council, 89124 Reggio Calabria, Italy; avilasi@ifc.cnr.it

**Keywords:** bronchial epithelial cells, cigarette smoke, oxidative stress, natural and synthetic antioxidants

## Abstract

Exposure of the airways epithelium to environmental insults, including cigarette smoke, results in increased oxidative stress due to unbalance between oxidants and antioxidants in favor of oxidants. Oxidative stress is a feature of inflammation and promotes the progression of chronic lung diseases, including Chronic Obstructive Pulmonary Disease (COPD). Increased oxidative stress leads to exhaustion of antioxidant defenses, alterations in autophagy/mitophagy and cell survival regulatory mechanisms, thus promoting cell senescence. All these events are amplified by the increase of inflammation driven by oxidative stress. Several models of bronchial epithelial cells are used to study the molecular mechanisms and the cellular functions altered by cigarette smoke extract (CSE) exposure, and to test the efficacy of molecules with antioxidant properties. This review offers a comprehensive synthesis of human in-vitro and ex-vivo studies published from 2011 to 2021 describing the molecular and cellular mechanisms evoked by CSE exposure in bronchial epithelial cells, the most used experimental models and the mechanisms of action of cellular antioxidants systems as well as natural and synthetic antioxidant compounds.

## 1. Introduction

The airway epithelium represents a critical interface with the external environment and is susceptible to injury from inhaled environmental pathogens and pollutants. Upper airways are covered by pseudostratified epithelium on the luminal mucosal surface, and are predominantly made of bronchial and bronchiolar epithelial cells including ciliated cells, goblet cells, secretory club cells, and basal progenitor cells, with less frequent neuroendocrine cells. To maintain tissue homeostasis, the airway epithelium is equipped with several defense mechanisms including complex and redundant antioxidant systems, antimicrobial defenses, mucus and mucociliary clearance mechanisms, and local sentinel immune cells. Age-related decline of these defense mechanisms is likely to participate in biochemical and physiological changes observed in the aging lung, and may contribute to the development of age-related chronic lung diseases including Chronic Obstructive Pulmonary Disease (COPD). COPD represents a major public health problem and is estimated to be the most common cause of chronic respiratory disease-attributable deaths, with 5.7% of total all-cause deaths [1].

Cigarette smoke (CS), the main risk factor for COPD, alters airway epithelial barrier function and increases oxidative stress promoting senescence and activation of pro-inflammatory pathways in the airway epithelium [2]. Genome-based failures (genomic instability, telomere attrition, epigenetic dysregulation), dysfunction of signaling pathways (nutrient-sensing aberrations, cell–cell communication anomalies, activation of inflammatory pathways, cell death, angiogenesis alterations) and organelles (mitochondrial abnormalities, loss of proteostasis) have been widely studied as hallmarks of oxidative stress-related cellular aging.

Oxidative stress occurs in both nucleated and non-nucleated cells [3,4] when endogenous antioxidant defenses are impaired and/or overwhelmed by the increased presence of reactive oxygen species (ROS). ROS, including superoxide anions (O_2_^−^), hydroxyl radicals (OH), and hydrogen peroxide (H_2_O_2_), generated by the transformation of oxygen through enzymatic and non-enzymatic reactions. ROS levels within the airways can derive from exogenous insults, as in the case of CS, or from endogenous sources such as cell injury and/or activated inflammatory and structural cells.

Besides representing an exogenous source of ROS, CS indirectly promotes oxidative stress by impairing the activity of key endogenous antioxidant systems, including the enzyme superoxide dismutase (SOD), glutathione, and nuclear factor erythroid 2-related factor 2 (Nrf2). The resulting increased oxidative stress causes aberrant activation of pro-inflammatory pathways, epigenetic modifications, such as DNA methylation and histone modification and altered expression of non-coding RNAs. Moreover, CS-induced oxidative stress causes lipid peroxidation, DNA damage and protein carbonylation, which in turn creates neoantigens responsible for the production of auto-antibodies. As a result, these events negatively impact several cellular functions including proliferation and survival, autophagy and mitochondrial activity. Antioxidant therapies are feasible approaches in alleviating oxidative stress- related airway epithelium impairment. In vitro cellular models of airway epithelial cells injured by cigarette smoke extract (CSE) represent a valid option to study the cellular and molecular events related to increased oxidative stress and to test new therapeutic approaches able to counteract CSE-mediated cell damage and tissue injury and that may be useful for the management of COPD patients. Of note, most of the knowledge herein reported can be expanded beyond the context of COPD as CS is a major risk factor for other chronic lung diseases, including asthma and idiopathic pulmonary fibrosis [5] as well as cardiovascular diseases [6].

This review summarizes our current understanding of the impact of increased oxidative stress in human bronchial epithelial cells exposed to CSE. Here, cellular models of bronchial epithelial cells, traditional and innovative methods for ROS detection, cellular and molecular events altered by CSE-induced oxidative stress, cellular antioxidant activities and the role of therapies with antioxidants are presented.

## 2. Materials and Methods

On 3 August 2021, we searched PubMed, Scopus, Web of Science and Embase, using combinations of Medical Subject Headings, explosion searches, and free keywords. The full search strategy is reported in the Supplementary Materials S1.

The criteria for study inclusion were: (1) human studies; (2) normal bronchial epithelial cells; (3) oxidative stress; (4) CSE; (5) presence of in vitro/ex vivo (i.e., primary cells) experimental models; (6) publication year from 2011 to 2021; (7) English language; (8) papers with available abstract. Exclusion criteria were: (1) animal-only studies; (2) studies without cellular models (e.g., papers dealing with histological analyses); (3) studies focusing on exposure to electronic-cigarette smoke or studies where cells were stimulated with single tobacco components (e.g., nicotine, benzopyrene, acrolein); (4) studies focusing on cancer cells, alveolar cells, or epithelial cells from other organs; (5) papers out of topic; (6) case reports, reviews, or conference abstracts.

The studies identified in the four databases were combined and duplicates were removed. Two reviewers (S.L.G. and E.P.) independently screened the articles for relevance based on titles and abstracts. Then, the full texts of the retrieved studies were evaluated by all the authors. In both screening phases, any disagreement was resolved through negotiation. The following information was extracted from the included articles: first author and publication year, study design, experimental procedures and main results. The extracted information was recorded in tabular format, and results were summarized.

## 3. Results

**Figure 1:**
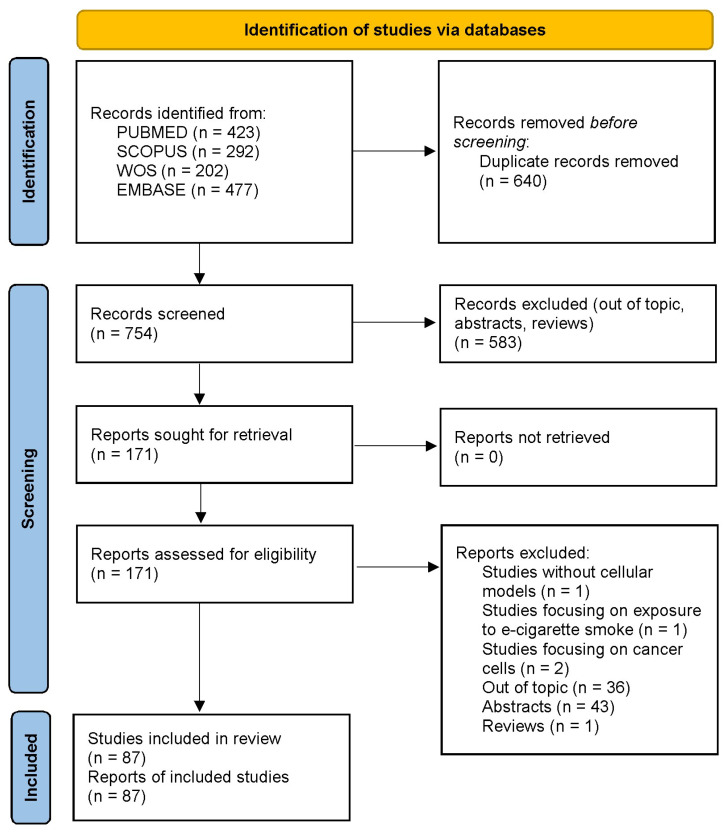
Flow diagram of the study selection process.

Figure 1 summarizes the study selection process. We identified 1394 articles through the four electronic databases. After the exclusion of duplicates, 754 articles were screened based on titles and abstracts, and 171 articles were identified as potentially eligible. Of them, 84 were excluded following full-text evaluation. Therefore, 87 articles were included in this review (Supplementary Materials S2).

### 3.1. Cellular Models of Bronchial Epithelium Exposed to CSE: Primary Cell Cultures and Cell Lines, 2D and 3D Models

Several cellular models are currently available to investigate the impact of CS on oxidative stress in bronchial epithelial cells, including primary cell cultures and cell lines, 2D and 3D models. Primary human bronchial epithelial cells (PBEC) can be isolated from bronchial brushing, lung biopsies and lung resections [7,8,9,10,11]. Independently from the source, the use of primary cells offers the advantage of studying cells from selected patient populations affected by respiratory diseases and consequently having increased risk for the selected inhaled toxicant [12]. As a drawback, if compared to cell lines, being directly isolated from human subjects, they are more difficult to obtain, lead to highly variable results and require a larger sample size to obtain significant statistics. In addition, culturing primary bronchial epithelial cells require expensive culture media and supplements.

For these reasons, immortalized human bronchial epithelial (HBE) cells are widely used and established as experimental models to study lung diseases [10,11,12,13,14,15,16,17,18,19,20,21,22,23,24,25,26].

Among the commercially available cell lines, the HBE cell line BEAS-2B (BEAS) has been used for over 30 years to model the pulmonary epithelium in the study of respiratory injury, wound healing and other experimental conditions. The BEAS cell line derives from epithelial cells obtained from normal human bronchial epithelium isolated from autopsy of non-cancerous lung tissue and then immortalized by transformation with an adenovirus 12-SV40 virus hybrid (Ad12SV40) [11,15,27,28,29,30,31,32,33,34,35,36,37,38,39,40,41,42,43,44,45,46,47,48,49,50,51,52,53,54,55]. One of the advantages offered by BEAS cells is that phenotype can be easily modulated by culture conditions [56]. This is why papers describing the use of BEAS often report different culture conditions [57,58,59,60,61,62,63]. As an example, exposure of BEAS to fetal bovine serum is associated with squamous differentiation, alterations in cytokine secretion and response to toxic substances [64,65,66].

The NL20 (CRL-2503) cell line is a SV-40 immortalized, a non-tumorigenic HBE cell line derived from normal bronchus [47]. The SV40 large T antigen-transformed 16HBE cell line (16HBE) is an HBE cell line isolated from a 1-year old male patient and immortalized with the origin-of-replication defective SV40 plasmid (pSVori-). It is a clonal diploid (2n = 6) cell line previously used to study the functional properties of bronchial epithelial cells in inflammation and repair processes. It is commonly chosen because it retains the differentiated morphology and function of normal airway epithelial cells, including cytokeratin expression, the ability to form tight junctions, and directional ion transport [67,68,69,70,71,72,73,74,75,76,77,78,79,80,81,82,83,84,85,86,87,88,89,90].

Both primary cells and cell lines can be grown in submerged culture as cell monolayer (2D model), or in three-dimensional models (3D model). 2D models, although easier to culture and less expensive, scarcely mimic the lung environment, as they lack the three-dimensional structure of the lung, inter-cell communications among different types of cells, the effects of the extracellular matrix and fail to undergo mucociliary differentiation. This impacts intracellular signaling, solute diffusion, and interaction with growth factors that result in altered responses compared to what occurs in the real lung [91].

Recently, there has been a growing interest in understanding the molecular and cellular events underlying the development of respiratory diseases in order to discover new therapeutic targets and develop new therapies. This, combined with the recognition of the limits of 2D cellular models, has led researchers to develop novel models able to mimic in vitro the respiratory system. To this purpose, a 3D air–liquid interface (ALI) cultures were developed in the late 1980s: a key feature is that they allow the basal surface of the cells to be in contact with the liquid culture medium, while the apical surface is exposed to air. To generate ALI cultures, cells are seeded onto the permeable membrane of a cell culture insert, which is initially supplied with culture medium covering both apical and basal compartments. After reaching confluence, cells are exposed to air by removing the medium in the apical compartment, thus creating a growth condition that drives cell differentiation into a polarized pseudostratified epithelium with a mucociliary phenotype. 3D models can be established using both primary cells and cell lines.

For example, ALI cultures have been established for human primary cells derived from the nasal epithelium as well as proximal and distal airway epithelium [92,93,94,95,96]. Among cell lines, NL20 are specially used for 3D cultures. In addition, 16HBE cells can be grown in an ALI model where they are able to form cilia [67]. The complexity of ALI cultures can be increased by including human fibroblasts, immunocompetent cells or extracellular matrix components such as fibronectin or collagen [7,8,9,39,97,98,99,100,101,102,103].

With regard to the stimulation with CS, the most common and established procedure to stimulate 2D models is the use of CSE that is prepared by smoking one (or more) cigarette for a given time (usually 5 min) into a given volume of PBS (usually one cigarette is smoked into 10 mL of PBS). This procedure generates a CSE-PBS solution which is filter-sterilized and considered to be 100% CSE.

Usually, the concentration of CSE is checked spectrophotometrically by measuring the absorbance at 320 nm [89]. Exposure of ALI cultures to CS can be performed using CSE or whole cigarette smoke (using smoking chambers).

### 3.2. Methods for Detecting Oxidative Stress

Several methods are available to measure oxidative stress in cellular models. Most approaches are based on the use of probes that become fluorescent after reacting with ROS, including H_2_O_2_, peroxyl radical (ROO·), nitric oxide (NO), and peroxynitrite anion (ONOO-). These probes are not selective for a specific chemical entity, although their use provides a measure of the oxidative status of the cells. ROS can be detected both in the extracellular medium and at an intracellular level. Detection of extracellular ROS can be performed using commercially available kits. As an example, H_2_O_2_ can be quantified using the chromo/fluorogenic probe Amplex Red or through colorimetric methods based on the reaction of H_2_O_2_ with titanous sulfate to form a yellow complex [13,17]. Other fluorescent dyes emitting fluorescence after reaction with ROS in cell supernatants exist and are mostly based on the dichlorodihydrofluorescin (DCFH).

Alternatively, intracellular ROS can be measured after staining the cells with a cell-permeable, ROS sensitive probe using flow cytometry, fluorescence microscopy or a plate reader. In this case, the dyes are non-fluorescent when reduced, but become highly fluorescent upon oxidation. The most widely used dye is the 2′,7′-dichlorofluorescin diacetate (DCFDA) that become oxidized into 2′,7′-dichlorofluorescein (DCF) by intracellular ROS [11,14,22,26,28,29,32,33,35,42,45,50,68,72,83,84,85,87,88,89,104]. Other commercially available fluorogenic probes are also available [13,24,69,80]. The advantage of using these fluorescent probes is that reagents for ROS detection can be applied to cells directly in the growth media. However, a drawback of these approaches is that several steps are required for proper staining and that cells die after staining. Similarly, it is possible to specifically measure ROS accumulated in mitochondria by using dyes that selectively targets mitochondria [12,33,34,35,40,78,105].

The oxidative status of the cell can also be evaluated by measuring reduced glutathione (GSH) levels, which are decreased under oxidative stress conditions. GSH is measured in cell lysates using spectrophotometry [25,26,28,31,33,41,53,84,85,89,97], liquid chromatography coupled to mass spectrometry (LC–MS) [97], enzymatic methods [51] and fluorescence [87]. Custom-made or commercially available kits can be used [42,44]. Kits are also available to detect GSH and its oxidized form, GSSG [37,41].

Oxidative stress can also be measured indirectly, by analyzing the degree of lipid peroxidation, a phenomenon that occurs in the presence of elevated concentrations of oxidants. Malondialdehyde (MDA) is one of the products of lipid peroxidation, and can be easily detected by colorimetric or fluorometric analysis [28,29,33,39,70,71]. Alternatively, it is possible to measure the levels of 4-hydroxynonenale (4-HNE) protein adducts by ELISA [22,24]. Oxidative stress is also responsible for protein carbonylation. The intracellular level of protein carbonyls can be detected either by ELISA [97] or immunoblotting assays [10]. Other products of oxidative stress (3-nitrotyrosine (3-NT), 8-isoprostane and 8-hydroxydeoxyguanosine) can be detected using enzymatic immunoassays [44,88,90].

Besides quantifying intra/extra-cellular ROS levels, the evaluation of enzymatic activities contributing to ROS generation, such NADPH oxidase (NOX) enzymes and myeloperoxidase, provides key information on the oxidative status of the cell. The activity of NADPH oxidases, responsible for the generation of the anion superoxide through the conversion of NADPH into NADP^+^, can be examined using an enzymatic assay and colorimetric detection [22]. Myeloperoxidase catalyzes the conversion of H_2_O_2_ and chloride (Cl^-^) to hypochlorous acid (HOCl) and can be detected by using enzymatic/colorimetric assays [13,29].

#### Innovative Methods for the Detection of Oxidative Stress

In recent years, electrochemical sensors have attracted growing attention for the detection of biomarkers of inflammation and oxidative stress. In an electrochemical sensor, the signal is generated from the redox reactions that take place on the electrode surface.

**Figure 2:**
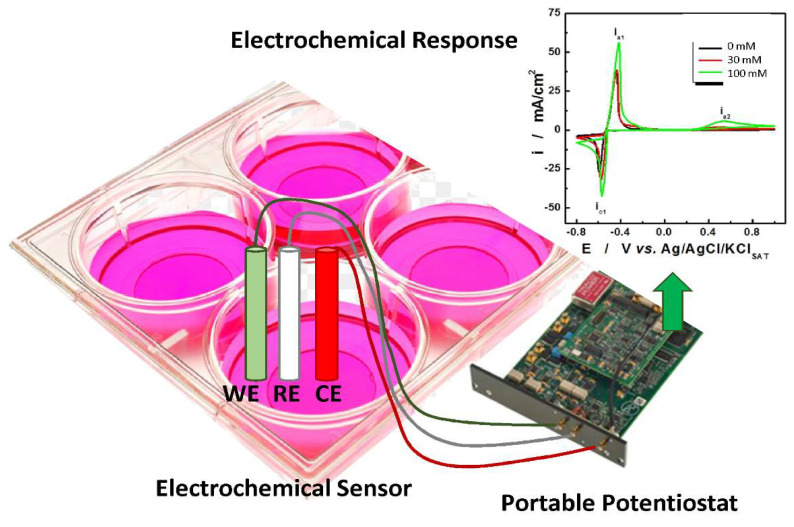
Real-time electrochemical detection of markers of oxidative stress in a culture plate.

Different electrochemical techniques, such as voltammetry, amperometry, or conduction/conductometric and impedance spectroscopy, can be used [106]. Electrochemical techniques possess several advantages such as low cost for both instrumentation (portable potentiostat can be easily assembled with very low-cost electronics, Figure 2) and materials, ease of use, even for unskilled personnel, and fast response times (less than 2 min to result) [107].

By making an appropriate choice of both the active material, for manufacturing the sensor, and the electrochemical technique used for the detection, it is possible to obtain sensors with high selectivity and sensitivity suitable for the detection of different markers of oxidative stress. In addition, recent works have also reported the possibility to use the electrochemical sensors direct in the culture plate for real-time monitoring of oxidative stress markers from living cells, without disturbing cell growth and without the need of medium sample collection for offline measurements [108,109]. In this way, the electrochemical sensor may have an easy-to-implement use in research laboratories, where the direct measurement of the markers of oxidative stress in cell supernatants can substitute current expensive and time-consuming biochemical and flow-cytometry-based approaches [110]. More importantly, in situ sensors can be used for monitoring changes of markers of oxidative stress levels upon different stimuli and collecting kinetic information on cellular release. Recently, wearable devices, implemented with wireless technology for signal transduction, have also been proposed for the direct and non-invasive detection in biological fluids (e.g., sweat, saliva and exhaled breath condensate) [111]. This may represent a valuable approach in telemedicine to easily monitor disease progression.

### 3.3. Molecular Alterations Induced by CS Exposure

**Figure 3:**
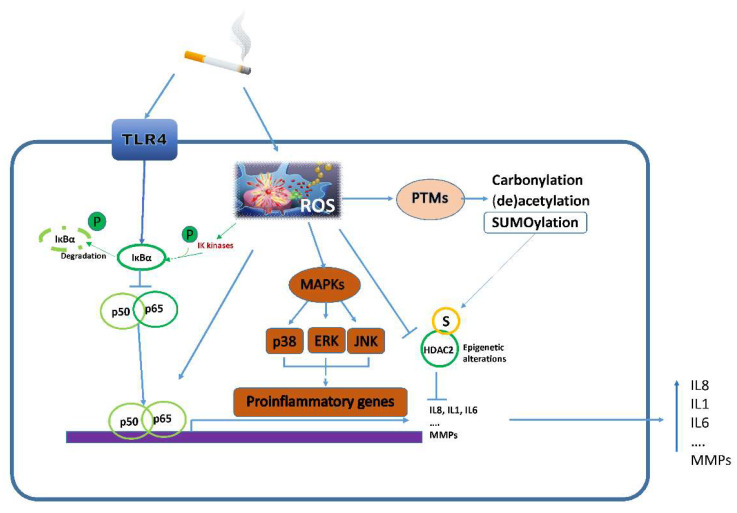
Molecular mechanisms altered by cigarette smoke in bronchial epithelial cells.

Prolonged exposure to CS promotes aberrant inflammatory responses in the airways, leading to tissue remodeling and subsequent reduction of airflow, which are the main features of COPD [16,27]. Oxidative stress generated by exposure to CS is responsible for exacerbated inflammation in the bronchial human epithelium [17,30]. Bronchial epithelial cells are the main source of cytokines, chemokines and adhesion molecules in the CS-exposed lung, modulating airway wall functionality and immune responses [27]. They respond to CSE by activating multiple inflammatory pathways, including mitogen-activated protein kinases (MAPKs), leading to the activation of extracellular signal-regulated kinases (ERK1/2), p38 and c-Jun N-terminal kinase (JNK) and the transcriptional factor nuclear factor kappa-light-chain-enhancer of activated B cells (NF-κB) (Figure 3).

Activation of these pathways is associated with increased expression and release of pro-inflammatory mediators including cytokines, chemokines and metalloproteinases (MMPs) [16,17,19,22,24,25,27,28,29,30,32,33,36,37,40,42,44,45,46,49,52,69,70,71,74,75,77,78,79,80,81,83,84,88,98,99,102,103,104,112,113], as well as altered regulation of non-coding RNAs (nc-RNAs) [70,71,74,102], occurrence of epigenetic modifications [72,78,79,89,114] and post-translational modifications (PTMs) [14,34,39,43,75,82,115,116].

#### 3.3.1. Inflammatory Pathways

The production of inflammatory mediators is controlled by several pathways, including NF-κB, Janus kinase/signal transducer and activator of transcription (JAK-STAT) and MAPKs. The toll-like receptor 4 (TLR4) plays a key role in transducing the proinflammatory effects of CS in the bronchial epithelium [84]. TLR4 expression is increased in response to CSE [84], and its activation triggers most of the CSE-induced proinflammatory pathways in HBE cells. In HBEs, NF-κB, the master regulator of transcriptional reprogramming during inflammation, was widely demonstrated to regulate the inflammatory response to CSE [17,22,24,27,32,36,42,45,70,77,78,98,99,112].

Additionally, stimulation of HBEs with CSE leads to activation of MAPKs, which is associated with proliferation, cell survival and motility, and cellular metabolism, and drives stress response and inflammation. More specifically, among MAPKs, activation of c-Jun N-terminal kinase (JNK), p38 and ERK1/2 has been widely described in relation to CSE-induced inflammation [22,27,29,36,46,49,53,74,77,83,86,88,98,102,103,104,113].

As is well known, the activation of these pathways leads to increased levels of pro-inflammatory cytokines such as TNF-α, IL-1β, IL-6, and IL-8. Of note, these cytokines are key mediators of CS-induced lung inflammation as well [17,19,22,24,25,27,28,29,30,32,33,36,37,40,42,44,45,46,69,70,71,74,75,77,78,79,80,81,83,90,98,99,102,104,112,113]. ROS, increased by CS, enhance the activation of the IκB kinase (IKK) complex (IKKβ, IKKα, and NF-κB essential modulator (NEMO), leading to the phosphorylation, ubiquitination and proteasomal degradation of IκB, in particular IκBα. This promotes the activation of the NF-κB/p65 complex, a process which requires a number of PTMs (phosphorylation, acetylation, glycosylation). Active NF-κB translocates to the nucleus, where it induces the transcription of inflammatory genes [17,22,24,27,32,36,42,45,70,77,78,98,99,112]. It has been reported that treatment of BEAS-2B with CSE 2.5% for 24 h increased NF-κB expression with a higher level of nuclear phospho-p65, and activation of JNK, p38 and ERK1/2. Such effects resulted in increased release of the pro-inflammatory cytokine IL-8 and decreased release of the anti-inflammatory IL-10. The increase of IL-8 derived both from NF-κB activation and MAPKs induction via cyclic AMP (cAMP)/protein kinase A (PKA). Moreover, stimulatory protein 1 (Sp-1) transcription factor, responsible for IL-10 expression, was inhibited by CSE-derived ROS [27,104]. A correlation between the peroxisome proliferator-activated receptor γ (PPARγ) and the NF-kΒ pathway has also been described [17]. PPARγ regulates the activation of the Glutathione peroxidase 3 (GPx3) responsible for ROS reduction. Repeated exposure of HBE to CSE inhibits PPARγ expression, resulting in increased ROS production, activation of NF-κB/p65 and higher levels of the inflammatory mediators TNF-α, IL-6 and IL-8 [17]. Additionally, a higher level of the pro-inflammatory cytokines TNF-α, IL-1β and IL-6, in turn, sustains NF-κB activation, supporting and perpetuating the inflammatory state [32]. Besides promoting cytokines and chemokines release, activation of MAPKs and NF-κB pathways by CSE induces the expression and release of MMPs, which contribute to airway tissue disruption [19,29,98].

Proteomic analysis of proteins secreted in the apical liquid surface of HBE ALI cultures showed that oxidative stress derived from repeated exposure to CS-induced the upregulation of MMP-9, MMP-10 and MMP-14 mainly via the EGFR/ERK1/2 signaling pathway [98]. The activation of ERK1/2 pathway was also related to increased mucus secretion, a well-known pathological condition characterizing COPD. In particular, in normal HBEs treated with 5% CSE and stimulated with the viral mimetic Poly(I:C), the transcription of MUC5AC was enhanced via the induction of the EGFR/ERK1/2 pathway [103].

#### 3.3.2. Non-Coding RNAs

Non-coding RNAs regulate gene expression through direct binding to their target mRNAs, leading to inhibition of translation. CSE induces the upregulation of several non-coding RNAs in bronchial epithelial cells, including miR-21, SCAL1, circRNA oxysterol binding protein like 2 (circOSBPL2) and circRNA HECT domain and ankyrin repeat containing E3 ubiquitin protein ligase 1 (circ-HACE1) [70,71,74,102]. miR-21 is a small non-coding RNA involved in the regulation of oxidative stress-dependent inflammatory reactions and cell proliferation. It has been reported that miR21 is increased in 16HBE cells, promoting the release of IL-8 and the activation of the ERK pathway.

Induction of miR-21 by CSE occurs via TLR4, and it is reverted by treatment with the antioxidant apigenin [74]. CircOSBPL2 and circ-HACE1 increase in the lung of smokers and COPD subjects [70,71]. In 16HBE cells exposed to CSE, circOSBPL2 and circHACE1 are upregulated and contribute to apoptosis, inflammation and oxidative stress by acting as a sponge for miR-193a-5p and miR-485-3p, respectively [70]. More specifically, binding of circOSBPL2 to miR-193a-5p leads to the upregulation of bromodomain containing protein 4 (BRD4), which is the mRNA target of miR-193a-5p; this mediates CSE-dependent responses [70]. Similarly, circHACE1 sponges miR-485-3p, therefore relieving the inhibitory effect of on its target mRNA, TLR4, leading to TLR4 upregulation, enhanced inflammatory reactions and cell death in response to CSE [71].

#### 3.3.3. Post-Translational Modifications

PTMs are reversible modifications that cells use to fine-tune the activity of proteins in response to intra- and extra-cellular signals. Exposure to CSE has been reported to induce a number of PTMs, including phosphorylation (addressed in Section 3.1), SUMOylation, carbonylation and acetylation [14]. SUMOylation is characterized by a covalent binding of the small ubiquitin-like modifier (SUMO) to its substrate. CSE increases the expression of SUMO1 and Ubc9, the conjugating enzyme required for SUMOylation in HBEs [14]. This is associated with increased SUMOylation of several target proteins including CYP1A1 and histone deacetylase (HDAC) 2 [14,75], leading to altered enzymatic activity and thus contributing to oxidative stress and inflammation.

Among oxidative stress-induced PTMs, protein carbonylation plays an important role in transducing CSE-dependent effects. As reported in Colombo et al. [115], exposure of 16HBE cell line to CSE increased the carbonylation of several proteins involved in primary metabolic processes, cell cycle and chromosome segregation, thus contributing to alteration of cell homeostasis. CSE affects the expression and function of several acetylase/deacetylase enzymes, such as the sirtuins Sirt3, Sirt4 and Sirt5, which are specifically located in mitochondria. In BEAS-2B bronchial epithelial cells, CSE reduces the expression and activity of Sirt3, a primary mitochondrial deacetylase that regulates mitochondrial function through the deacetylation of several metabolic and respiratory enzymes. This is associated with a reduction of the antioxidant enzyme manganese superoxide dismutase (MnSOD) and leads to the increase of mitochondrial ROS generation and cell damage [34]. Regulation of Forkhead box class O 3a (FoxO3) depends also on Sirt1, a deacetylase normally localized in the cytoplasm of HBEs (both primary and cell lines). Regulation of Sirt1 activity occurs via a dynamic shuttling between cytoplasm and nuclei. Treatment with CSE induces PI3K-dependent Sirt1 nuclear shuttling in BEAS2B and primary HBE cells together with FoxO3 nuclear translocation, leading to the upregulation of several antioxidant genes, including heme oxygenase 1 (HO-1) [39]. Of note, in the same cell type nuclear, Sirt1 is reduced during chronic exposure to CSE or in COPD subjects [39]. Among possible mechanisms, it has been reported that neutrophil elastase (NE), which is increased in the lung of COPD subjects, can enter primary bronchial epithelial cells and cleave Sirt1, therefore reducing Sirt1 protein levels [43]. A reduction of Sirt1 activity and nuclear localization has been reported in 16HBE exposed to CSE. This is associated with a reduction of nuclear FoxO3 and increased senescence [82].

#### 3.3.4. Epigenetic Alterations

Epigenetic alterations include chromatin remodeling, DNA methylation and PTMs of histone proteins. Several reports have shown that exposure of bronchial epithelial cells to CSE causes a number of epigenetic modifications [72,78,79,89,114]. More specifically, in 16HBE, exposure to CSE downregulates MECP2 (Methyl-CpG-binding protein 2) and upregulates cytochrome family 1 subfamily B polypeptide 1 (*CYP1B1*), a member of the CYP1 family gene whose transcription is strongly modulated by DNA methylation of the promoter site. Increased methylation of *CYP1B1* promoter has been observed in CSE-exposed 16HBE [72]. Of note, CSE-induced cell injury and increased methylation of *CYP1B1* promoter were reversed by overexpression of MECP2 [72]. Concerning histone modification, exposure of 16HBE cells to CSE decreased HDAC3 activity/expression and HDAC2 activity, contributing to steroid resistance [78,79,89]. Conversely, CSE increased the expression of the histone acetyl transferase (HAT) p300/CBP [79].

### 3.4. Impact of CS on Cellular Homeostasis

**Figure 4:**
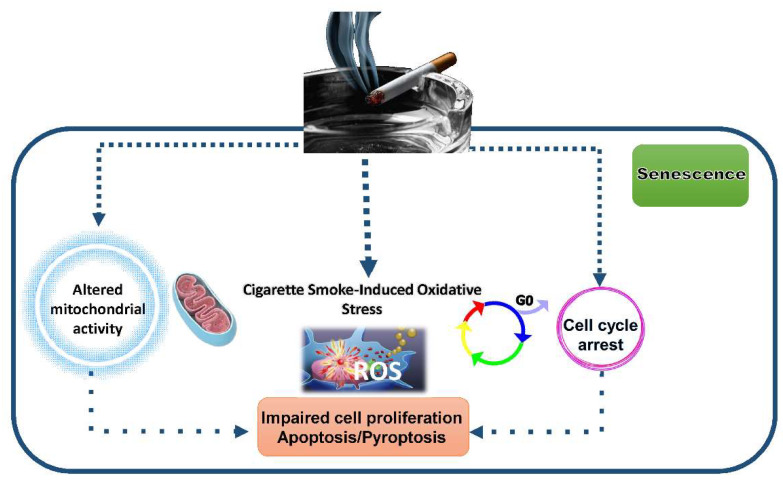
Impact of CS on bronchial epithelial cell physiology.

CS contains nearly 5000 reactive chemicals including ROS, lipophilic substances, mutagens, heavy metals and carcinogens. Exposure of bronchial epithelial cells to CS promotes oxidative stress, inflammation and profound changes in the cell physiology via transcriptional and post-transcriptional mechanisms. Altered mitochondrial function, impairment of autophagy, dysregulation of cell cycle control and cell proliferation, the imbalance between pro-survival and death pathways, and senescence are among the effects caused by exposure to CSE (Figure 4).

#### 3.4.1. Impact of CSE on Autophagy, Mitophagy and Mitochondrial Activity

Eukaryotic cells, including bronchial epithelial cells, remove unnecessary or dysfunctional components through a lysosome-dependent regulated mechanism named autophagy [38]. Functional autophagy is characterized by the formation of double-membraned vesicles called autophagosomes. During autophagy, the cytosolic LC3B-I form is converted into the lipidated LC3B-II form associated with autophagosomal membranes. LC3B-II is a ubiquitin-like protein, considered an autophagy marker as it exerts a crucial role in autophagosome maturation. LC3B-II becomes incorporated into the autophagosome membrane and is responsible for binding to p62, a cargo adaptor protein used to identify ubiquitinated proteins that are bound for autophagic degradation. During autophagic flux, both proteins are subject to degradation in autolysosomes. Mitochondria homeostasis is regulated by mitophagy, the selective degradation of mitochondria by autophagy [117]. In bronchial epithelial cells exposed to CSE, altered mitophagy, associated with mitochondrial fragmentation and impaired fusion/fission regulation, is observed together with the reduction of Miro1 and Pink1 [12], two proteins with a relevant role in mitophagy. This is associated with altered mitochondrial function, through disruption of mitochondrial membrane potential (MMP) and increased mitochondrial ROS production [12]. In addition, CSE increased the expression and mitochondrial translocation of p66Shc, a key regulator of mitochondrial function, promoting mitochondrial ROS generation and oxidative stress. Consistently, p66Shc siRNA significantly attenuated mitochondrial dysfunction and cell injury in response to CSE [40].

Since mitochondrial respiration is a major source of cellular ROS, the strong impact that CSE has on mitochondrial activity significantly contributes to the observed oxidative damage [34]. Several reports have shown that CSE alters autophagy/mitophagy in bronchial epithelial cells by affecting the expression and the activity of several enzymes with a key role in these processes [12,31,105]. As an example, unc-51-like kinase 1 (ULK1) and mitogen-activated protein kinase 15 (MAPK15), the atypical member of the MAPK family, are involved in the signal transduction of conventional autophagy. Of note, these two enzymes are involved in mitophagy during the pathogenesis of COPD and contribute to CSE-induced mitophagy and mitochondrial oxidative injury in airway epithelial cells.

Therefore, the inhibition of MAPK15-ULK1 signaling pathway might improve the function of airway epithelial cells and may represent a novel therapeutic strategy for COPD [29,105].

#### 3.4.2. Impact of CSE on Senescence and Cell Death

Eukaryotic changes in mitochondrial activity and homeostasis occur during long-term exposure to CSE support aging processes [118,119].

Chronic inflammation and cellular senescence, also known as *inflammaging*, are deeply involved in the pathogenesis of premature lung aging, which is considered an important contributing factor in driving COPD. CS promotes accelerated epithelial cell senescence within the lung. Cell senescence is characterized by a loss of replicative potential and increased beta galactosidase activity. In bronchial epithelial cells, CSE reduces short- and long-term cell proliferation, increases β-galactosidase activity and decreases the expression and activation of anti-aging Sirt1, a NAD^+^-dependent histone deacetylase [82]. Sirt1 protects against senescence via regulating FoxO3 activity, p53, p-21, NF-κB, histones and a variety of proteins involved in DNA damage repair [29,97]. Deacetylation of FoxO3 by Sirt1 favors the expression of antioxidant genes and negatively regulates pro-survival genes, such as survivin.

Survivin is essential in protecting cells from entering apoptosis, controlling cell growth and contributing to senescence regulation in a stress environment context [120]. Altered activity of the Sirt1/FoxO3 axis in bronchial epithelial cells exposed to CSE leads to increased expression of survivin [82]. As a consequence, CSE confers the resistance of bronchial epithelial cells to apoptosis and promotes senescence.

Bronchial epithelial cells senescent phenotype is associated with higher pro-inflammatory properties. Secretion of IL-8, a hallmark of the senescence-associated secretory phenotype (SASP) and a potent chemokine for neutrophils, is increased upon TLR4 activation and ERK1/2 activation by CSE [121]. The release of NE increases CSE-induced IL-8 production but reduces vascular endothelial growth factor (VEGF) levels, promoting its degradation in bronchial epithelial cells [46]. The decreases of VEGF expression and release caused by CS exposure contributes to CS-induced alteration of lung homeostasis, such as apoptosis of epithelial cells in the emphysematous lung, loss of microvasculature in lung parenchyma of COPD and altered macrophage functions [46,122,123,124].

Besides affecting apoptosis and promoting cell senescence, it has been reported that CSE promotes other inflammatory forms of cell death, such as the pyroptosis or other forms of caspase-dependent cell deaths [12,14,15,27,28,30,31,32,33,34,68,69,72,73,105]. Pyroptosis is a type of pro-inflammatory cell death promoted by caspase-1 activation and characterized by cell lysis and interleukin IL-1β release. Growing evidence suggests that increased pyroptotic cell death may contribute to CS-induced lung inflammation. Whether the NLRP3 inflammasome participates in CS-dependent inflammatory events in bronchial epithelial cells is still a matter of debate [125].

### 3.5. Cellular Antioxidant Responses

Bronchial epithelial cells are equipped with multiple antioxidant defense systems to counteract CSE-induced oxidative stress and maintain cellular homeostasis. Total antioxidant status (TAS) and total oxidant status (TOS) of a cell can be measured by performing biochemical assays. Briefly, the TAS method depends on the ability of antioxidants present in the sample to inhibit ABTS radical cation formation from the oxidation of ABTS (2,2′-azino-di-3-ethylbenzthiazoline-6-sulfonic acid) [18]. Several commercial kits are available that measure the antioxidant capacity of the cell based on the principle of oxidation inhibition [38,50]. Antioxidant capacity and free radical scavenging potential of a cell can also be measured spectrophotometrically using the ferric reducing antioxidant power (FRAP) assay and the reduction of blue tetrazolium (NBT), respectively [19].

The TOS method is based on the capacity of oxidants in the sample to oxidize the ferrous ion to ferric ion and the measurement of the ferric ion by xylenol orange [18].

#### 3.5.1. Enzymatic Antioxidant Systems

The well-recognized enzymes able to directly neutralize intracellular ROS include the enzyme SOD, that converts superoxide to H_2_O_2_, the catalases, converting H_2_O_2_ to H_2_O, and thioredoxin (TRX)- or glutathione-dependent peroxidases (GSH-Px), that catalyzes the reduction of hydroperoxides (e.g., H_2_O_2_) to H_2_O via oxidation of GSH into GSSG. SOD or catalase enzymatic activities can be measured using commercially available colorimetric assays [11,25,28,29,31,32,34,44,45,70,71,77,83,112]. In bronchial epithelial cells exposed to CSE, the levels of SOD [25,29,30,31,34,39,42,45,77,112], catalase [25,31,42,77] or GSH-Px [44,77,87] are significantly reduced.

TRX and glutaredoxin (GRX) are the major thiol-dependent antioxidant systems in mammalian cells and maintain protein cysteine residues in the reduced state. They are therefore key systems for maintaining redox homeostasis. In addition, TRX downregulates the activities of many oxidative stress-sensitive transcription factors such as NF-κB, Nrf-2 and p53 [126]. The GRX and TRX reciprocally act as recovery systems so that, if one system loses its electron transfer function, the other replaces this function. The TRX system is composed of NADPH, thioredoxin reductase (TrxR) and TRX. Mammalian cells possess two TRX systems, the cytosolic Trx1 and the mitochondrial Trx2. In the cytosol, TrxR catalyzes the reduction of the redox-active disulfide in TRX by NADPH. Reduced TRX is a powerful defense against oxidative stress. A similar action is exerted by the GRX system which comprises GRX, glutathione, glutathione reductase (GR) and NADPH. Moreover, within the cytosol and mitochondria, TRX reducing methionine sulfoxide reductases promotes the repair of oxidized proteins [126].

NADPH, the coenzyme required by TrxR and GR, is produced through the oxidative pentose phosphate pathway and through the activities of different dehydrogenases located both in the cytosol and in the mitochondria, including NAD(P)H quinone dehydrogenase 1 (NQO1), aldehyde dehydrogenase-1-L1 (ALDH1L1; cytoplasm) and aldehyde dehydrogenase-1-L2 (ALDH1L2; mitochondria). Of note, CSE reduces NQO1in bronchial epithelial cells [25,26,42,112]. Finally, peroxiredoxins (PRDXs) are a ubiquitously expressed family of small (22–27 kDa) peroxidases that catalyze the reduction of hydroperoxides and peroxynitrite.

#### 3.5.2. Non-Enzymatic Antioxidant Activities

Reduced glutathione (GSH) is often referred to as the most important non-enzymatic antioxidant in cells. It is concentrated in the epithelial lining fluid and plays a critical role in maintaining intracellular redox status, in addition to detoxifying compounds via conjugation reactions catalyzed by GSH S-transferase (GST). The ratio of reduced GSH to oxidized GSH (GSSG) is an indicator of cellular health, with reduced GSH constituting up to 98% of cellular GSH under normal conditions. In a resting cell, the molar GSH:GSSG ratio exceeds 100:1, while, under oxidative stress conditions, this ratio has been demonstrated to decrease to values of 10:1 and even 1:1. Upon increased oxidative stress, GPx and PRDX6 catalyze the reduction of H_2_O_2_ by GSH to oxidized glutathione (GSSG) and water. Accumulation of GSSG is potentially cytotoxic, so cells normally maintain a high activity of GR to restore the normal ratio of GSH:GSSG. In bronchial epithelial cells exposed to CSE, GR [31,44,87], GSH or GSH:GSSG ratio [25,28,31,42,44,53,77,84,85,87] are reduced.

The expression of γ-glutamylcysteine synthetase ( *GCLC*), coding for the enzyme responsible for the synthesis of GSH, is regulated at a transcriptional level by the antioxidant responsive elements (AREs). The protective functions of GSH involve enzymatic as well as non-enzymatic processes. The enzymatic processes include the regeneration of GSH-PXs, which detoxify lipid hydroperoxides to non-toxic hydroxy fatty acids, the involvement in GST reactions required for detoxification of xenobiotics or oxidative stress products, and the glutathionylation/deglutathionylation reactions catalyzed by the GRX system. GSH-PXs are reduced in bronchial epithelial cells exposed to CSE [44,77,87].

In addition, GSH supports the non-enzymatic regeneration of alpha-tocopherol, preventing lipid peroxidation in cellular membranes and directly neutralizing the superoxide anion radical. GSH plays a key role in maintaining oxidant-induced lung epithelial cell function and in the control of pro-inflammatory processes.

Nrf2 is a redox-sensitive transcription factor that acts as a central regulator of phase II detoxification systems and antioxidant response. Kelch-like ECH-associated protein 1 (Keap1) is the key regulator of Nrf2. Keap-1 is an adaptor for a Cul3-based E3 ligase that conjugates ubiquitin to Nrf2, promoting its proteasomal degradation.

Oxidative stress induces the oxidation of cysteine residues and promotes a conformational change of Keap-1 leading to the detachment of Nrf2 from Keap-1. This event prevents the ubiquitination and the proteasomal degradation of Nrf2 and allows the nuclear migration of Nrf2. Nuclear Nrf2 binds to AREs and activates a strong antioxidant transcriptional program. After the resolution of the stressing event, Keap1 can enter the nucleus and shuttle Nrf2 back to the cytosol.

The genes controlled by AREs encode several group of enzymes: (a) phase I antioxidant enzymes such as SODs, GPx, GR and γ-GCS; (b) phase II detoxifying enzymes such as GST, NADP(H):quinone oxidoreductase-1 (NQO1), glutamate-cysteine ligases (GCLM/GCLC); (c) phase III xenobiotic transporters such as multidrug resistance protein 1 (MRP1); (d) other stress response proteins such as HO-1. Furthermore, Nrf2 can activate PPARγ, thus controlling adipogenesis, but it can also potentiate the antioxidant responses inducing the expression of GPx in bronchial epithelial cells [17]. Upon oxidative stress induced by CSE, Nrf2 activity is compromised [20,26,28,30,33,44,50,53,54,85,89,112]. The levels of Nrf2 can also be affected by epigenetic events induced by oxidative stress. Physiologically, HDAC2 is associated with Nrf2 and prevents its degradation probably by deacetylation of lysine residues [54]. HDAC becomes oxidized and moves from the nucleus to the cytosol. Trx1 can reduce the oxidation processes, thus shuttling back HDAC to the nucleus.

BTB Domain and CNC Homolog 1 (Bach1) functions primarily as a transcriptional suppressor since Bach1-Maf heterodimers inhibit the transcription of many oxidative stress-response genes, including *HO-1* and NADPH quinone oxidoreductase 1 (*NQO1*) by binding to Maf recognition elements (MAREs) in the gene promoters (Chang, Wen-Hsin 2017; Boylston 2014). Upon exposure to oxidative stress, Bach1 translocates into the cytoplasm, while Nrf2 dissociates from its cytoplasmic inhibitor, Keap-1, enters the nucleus and binds to MAREs. Bach1 competes with Nrf2 to bind to the MAREs and to regulate the oxidative stress-response genes. In bronchial epithelial cells, the acute exposure to CS causes nuclear translocation of Nrf2 together with a decrease in nuclear Bach1 allowing an increased HO-1 mRNA expression [8].

**Figure 5:**
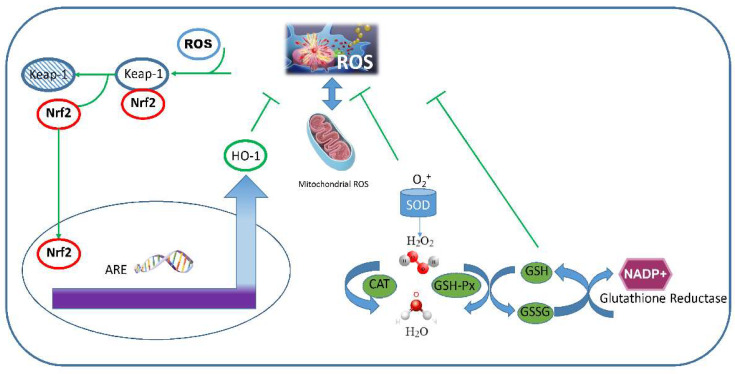
Anti-oxidant mechanisms of bronchial epithelial cells.

HO-1 is an Nrf2-controlled enzyme that plays a key role in cellular adaptation to injury and in maintaining cellular homeostasis. The enzymatic activity of HO-1 requires molecular oxygen (O_2_), NADPH and cytochrome p-450 reductase as the electron source and generates several products including carbon monoxide (CO). CO can influence mitochondrial function and/or cellular signal transduction programs which culminate in anti-apoptotic, anti-proliferative and anti-inflammatory events. In particular, CO downregulates TLR-mediated pro-inflammatory signals negatively interfering with p38, NF-κB and inflammasome activation. At the same time, CO promotes anti-inflammatory responses by activating PPARγ and promoting the synthesis of pro-resolving mediators. The expression and activity of HO-1 is compromised in bronchial epithelial cells exposed to CSE [21,25,26,42,44,85,89,112]. The main antioxidant activities of bronchial epithelial cells are summarized in Figure 5.

#### 3.5.3. Natural and Synthetic Compounds Able to Counteract CS-Induced Oxidative Stress

A huge number of natural compounds, as well as synthetic-derived molecules with antioxidant activities in bronchial epithelial cells, have been reported (Table 1 and Table 2). These compounds are able to counteract CS-dependent oxidative damage and may be considered for the treatment of chronic inflammatory lung diseases characterized by elevated oxidative stress.

## 4. Conclusions

The bronchial epithelium is at the front line to defend the human lung from invading pathogens and inhaled toxic agents. Bronchial epithelial cells are therefore well equipped with robust and redundant antioxidant and detoxifying systems to counteract insults deriving from the external environment. Nevertheless, when the exposure to oxidants is very strong and prolonged in time (i.e., chronic exposure), endogenous antioxidant systems are overwhelmed and can no longer maintain cellular homeostasis. This generates a condition known as “oxidative stress”, where a number of pro-inflammatory pathways become activated, generating a feed-forward loop difficult to resolve and that most often results in chronic inflammation and tissue injury. CS represents a major risk factor for most chronic inflammatory diseases, especially lung diseases, such as COPD.

The use of experimental models representative of the lung epithelium insulted by CS provides the tools for the study of molecular mechanisms underlying oxidative stress-related diseases, in order to discover new therapeutic targets and develop new drugs. Promoting endogenous antioxidant systems, as well as relieving oxidative insults through natural or synthetic antioxidant compounds, may be useful strategies for the development of new therapies. In this scenario, the possibility of real-time assessing oxidative stress in readily available biological fluids is becoming of great importance to monitor disease progression and response to therapies. In this respect, the development of innovative electrochemical sensor-based detection systems is paving the way for improved diagnosis, telemedicine and targeted therapeutic approaches.

Finally, stopping or avoiding smoking cigarettes, good nutritional status with a proper intake of natural antioxidants and regular physical activity remain the three fundamental factors to counteract oxidative stress and mitochondrial damage and represent the best primary prevention strategy to preserve human health [127].

## Figures and Tables

**Table 1:** Natural antioxidant compounds.

Name	Effects	References
Eucalyptol curcumin	↓apoptosis ↓oxidative damage ↓inflammation ↑antioxidant response (GSH, Nrf2)	[28]
Naringenin	↓apoptosis ↓oxidative damage (SOD, NQO1 and HO-1) ↓inflammation ↑antioxidant response	[112]
Dendrobiumofficinale polysaccharides	↓mucus secretion and viscosity	[7]
Alantolactone	↓inflammation (NFkB) ↑antioxidant response (Nrf2-HO-1 pathway)	[30]
Thymoquinone	↑antioxidant response (SOD, CAT, GR, GSH) ↓mitochondrial dysfunction	[31]
Epigallocatechin gallate	↓oxidative stress (ROS) ↓inflammation (NF-kB)	[24]
Chrysophanol	↓cell apoptosis (Bax, caspases) ↓oxidative stress (CYP1A1) ↓ER stress	[73]
Ginsenoside Rb3	↓inflammation (Il-8, TNF-alpha, p38 and NFkB) ↑antioxidant responses (SOD, catalase, GSH, GPx)	[77]
Andrographolide	↑autophagy (LC3B-II ↓oxidative stress (ROS) ↑antioxidant responses (Nrf2 and p62-Nrf2 positive feedback)	[38]
Oroxylin A	↑antioxidant responses (Nrf2, GSH, GR, GPx, HO-1)	[44]
Luteolin	↑antioxidant responses (GSH, Nrf2, NQO1 and HO-1) ↓oxidative stress (ROS) ↓cell apoptosis (caspases 3, 8 and 9)	[26]
Wedelolactone	↑antioxidant responses (SOD, catalase, GSH, Nrf2, NQO1 and HO-1)	[25]
Sesaminol	↓inflammation (IL-8, IL-6) ↓apoptosis ↓oxidative stress (ROS) ↑antioxidant responses (SOD and catalase)	[45]
17-0xo-DHA	↓oxidative stress (ROS) ↑antioxidant responses (GSH, Nrf2, HO-1)	[85]
Resveratrol	↑antioxidant responses (Nrf2)	[23]

**Table 2:** Synthetic antioxidant compounds.

Name	Effects	References
Beclomethasone dipropionate loaded into nanoparticles into liposomes and hyalurosomes modified with mucin	↓oxidative stress (ROS)	[68]
Beclomethasone+ formoterol	↓oxidative stress (ROS) ↓inflammation (STAT-1)	[88]
Fluticasone propionate±formoterol	↓inflammation (HDAC2, ERKSTAT-1)	[78]
Fluticasone propionate loaded in nanostructured lipid carriers	↓oxidative stress (ROS) ↑antioxidant response (GSH)	[84]
Dexamethasone	↓oxidative stress (ROS) ↑antioxidant responses (SOD, catalase) ↓inflammation (NF-kB, COX-2)	[32]
Sulforaphane and Sulforaphane N-acetylcysteine	↓oxidative stress (ROS) ↑antioxidant responses (Nrf2) ↓inflammation (ERK/JNK) CSE exposure	[33]
N-Acetyl-cysteine and Curcumin, Vitamin B2, Carnitine	↓inflammation (IL-1β, IL-6, TNFα) ↑antioxidant responses (Nrf2, HO-1and PPAR-γ)	[80]
Cardiac glycosides (Strophanthidin, digoxin, and digoxigenin)	↑authophagy (p62 and bicaudal D1).	[15]
Carbocysteine	↓oxidative stress (ROS) ↑antioxidant responses (GSH, Nrf2, HO-1, GSH-Px2 and 3, GR and glutamate-cysteine-ligase) ↓inflammation (HDAC-2, IL-8) Senescence (Sirt-1/FoxO3 axis)	[75,87,89]
Carbocysteine and beclomethasone	↓inflammation (pCREB, IL-1 mRNA and neutrophil chemotaxis)	[79]
Tiotropium and Tiotropium and long acting b2 agonist	↓inflammation (IL-8) ↓oxidative stress (ROS)	[90,104]
Selegiline	↓inflammation (IL-8) ↓oxidative stress (ROS) ↑antioxidant responses (GSH/GSSG ratio, SOD, catalase, Nrf2, Bach1, HO-1)	[42]
Ketanserin	↓inflammation (p38, ERK1/2, IL-8) ↑antioxidant responses (Nrf2)	[53]

## Data Availability

Not applicable.

## Supplementary Materials

The following supporting information can be downloaded at: https://www.mdpi.com/article/10.3390/ijms23031770/s1.
