# Supplementary material for: Cellular and Molecular Signatures of Oxidative Stress in Bronchial Epithelial Cell Models Injured by Cigarette Smoke Extract
Source: Int J Mol Sci. 2022 Feb 4;23(3):1770. doi: 10.3390/ijms23031770 (PMC8836577; doi:10.3390/ijms23031770)
Supplement: Supplementary file 1 [file ijms-23-01770-s001.zip › Supplementary Material S1 - Search strategy.pdf]

Cellular and molecular signatures of oxidative stress in bronchial epithelial cell models injured by cigarette smoke extract: Search Strategy.

PUBMED

(“Oxidative Stress”[Mesh] OR “oxidative stress*”[TIAB]) AND (“Epithelial Cells”[Mesh] OR “alveolar epithelial cell*”[TIAB] OR “lung epithelial cell*”[TIAB] OR “airway epithelial cell*”[TIAB] OR “bronchial epithelial cell*”[TIAB]) AND (“Smoke”[Mesh] OR smok*[TIAB] OR cigarette*[TIAB] OR tobacco[TIAB]) AND y_10[Filter] AND english[Filter] AND fha[Filter] NOT (“animals”[Mesh] NOT “humans”[Mesh])

SCOPUS

TITLE-ABS-KEY(“oxidative stress*”) AND TITLE-ABS-KEY(“alveolar epithelial cell*” OR “lung epithelial cell*” OR “airway epithelial cell*” OR “bronchial epithelial cell*”) AND TITLE-ABS-KEY(smok* OR cigarette* OR tobacco) AND PUBYEAR AFT 2010 AND LANGUAGE(english)

WEB OF SCIENCE

(TI=(“oxidative stress*”) OR AB=(“oxidative stress*”)) AND (TI=(“alveolar epithelial cell*” OR “lung epithelial cell*” OR “airway epithelial cell*” OR “bronchial epithelial cell*”) OR AB=(“alveolar epithelial cell*” OR “lung epithelial cell*” OR “airway epithelial cell*” OR “bronchial epithelial cell*”)) AND (TI=(smok* OR cigarette* OR tobacco) OR AB=(smok* OR cigarette* OR tobacco)) AND PY=(2011-2021) AND LA=(English)

EMBASE

('oxidative stress'/exp/mj OR 'oxidative stress*':ab,ti) AND ('epithelium cell'/exp/mj OR 'alveolar epithelial cell*':ab,ti OR 'lung epithelial cell*':ab,ti OR 'airway epithelial cell*':ab,ti OR 'bronchial epithelial cell*':ab,ti) AND ('smoke'/exp/mj OR smok*:ab,ti OR cigarette*:ab,ti OR tobacco:ab,ti) AND [english]/lim AND [embase]/lim AND [2011-2021]/py NOT ([animals]/lim NOT [humans]/lim)
